# Low level of alcohol drinking among two generations of non-Western immigrants in Oslo: a multi-ethnic comparison

**DOI:** 10.1186/1471-2458-12-535

**Published:** 2012-07-23

**Authors:** Ellen J Amundsen

**Affiliations:** 1 SIRUS/Norwegian Institute for Alcohol and Drug Research, P.O. Box 565 Sentrum, Oslo, NO-0105, Norway

**Keywords:** Alcohol use, Social interaction, Immigration, Ethnicity, Muslim, Gender

## Abstract

**Background:**

Alcohol drinking is a risk factor for harm and disease. A low level of drinking among non-Western immigrants may lead to less alcohol-related harm and disease. The first aim of this study was to describe frequency of drinking in two generations of immigrants in Oslo, contrasting the result to drinking frequency among ethnic Norwegians. The second aim was to study how frequency of drinking among adult immigrants was associated with social interaction with their own countrymen and ethnic Norwegians, acculturation, age, gender, socioeconomic factors and the Muslim faith.

**Method:**

The Oslo Health Study (HUBRO) was conducted during the period 2000 to 2002 and consisted of three separate surveys: a youth study (15-16-year-olds, a total of 7343 respondents, response rate 88.3%); adult cohorts from 30 to 75 years old (18,770 respondents, response rate 46%); the five largest immigrant groups in Oslo (aged 20–60 years, a total of 3019 respondents, response rate 39.7%). Based on these three surveys, studies of frequency of drinking in the previous year (four categories) were conducted among 15-16-year-olds and their parents’ generation, 30-60-year-old Iranians, Pakistanis, Turks and ethnic Norwegians. A structural equation model with drinking frequency as outcome was established for the adult immigrants.

**Results:**

Adults and youth of ethnic Norwegian background reported more frequent alcohol use than immigrants with backgrounds from Iran, Turkey and Pakistan. Iranians reported a higher drinking frequency than Turks and Pakistanis. In the structural equation model high drinking frequency was associated with high host culture competence and social interaction, while high own culture competence was associated with low drinking frequency. Adult first-generation immigrants with a longer stay in Norway, those of a higher age, and females drank alcohol less frequently, while those with a higher level of education and work participation drank more frequently. Muslim immigrants reported a significantly lower drinking frequency than non-Muslims, although this did not apply to Iranians.

**Conclusions:**

The existence and growth in Western societies of immigrant groups with low-level alcohol consumption contributed to a lower level of consumption at the population level. This may imply reduced drinking and alcohol-related harm and disease even among ethnic Norwegians.

## Background

Alcohol use has been ranked high as a risk factor for death and loss of healthy life years, as well as being a source of personal and social harm [1-3]. Policies and interventions exist that can reduce alcohol use, but there is still a need to understand more about why alcohol use and abuse change in populations and how alcohol-related harm can be reduced [4].

Studies in Europe have generally found that immigrants from non-Western countries tend to drink less or less often than the host population. In the Netherlands, Turkish and Moroccan immigrants reported less alcohol use than the Dutch population in both the first and second generation, and their alcohol consumption did not converge towards the higher rates in the host population [5,6]. In another study from the Netherlands mono-ethnic immigrants in secondary schools from the Antilles and Surinam were also included in addition to Turks and Moroccans, all reporting a lower prevalence of drinking than the Dutch students [7]. In the United Kingdom, African Caribbeans have reported lower alcohol consumption than their white counterparts in studies from 1986 to 1995 [8]. Asian Muslims reported a very low level of alcohol consumption in the United Kingdom [9]. Muslim 15-16-year-olds in England, as well as Hindus and Sikhs in the same age group, reported a far lower level of consumption than white adolescents [10]. In Germany, among 9^th^ graders, the largest immigrant group (Turks) reported less than half the level of binge drinking in the previous four weeks than those of German descent. The drinking behaviour of the second largest group of immigrants in Germany (from the former Soviet Union) was similar to those of German descent. Otherwise adolescents from Islamic countries living in Germany had a lower lifetime prevalence of drinking than German and Western European adolescents, with the exception of students from Iran [11]. In a study from Oslo, Norway, 15-16- year-olds with an ethnic Norwegian background reported a higher frequency of drinking than their age-peers from Pakistan, Somalia, Turkey and Morocco where Islam was the predominant religion. The frequency of drinking among youth from Iran, another country where Islam is the predominant religion, was also lower than among Norwegians, but not as low as among youth from the Islamic countries already mentioned. A large proportion of youth from Vietnam had tasted alcohol, but fewer drank often and fewer got drunk compared to ethnic Norwegians [12]. Albanians in Florence, Italy, reported more drinking, however, than Albanians in the home country and among Italians [13]. Also, in a study of recent Hispanic immigrants to Spain over the age of 15, there were no significant differences between the previous 12 months’ drinking among immigrants compared to the native population. Almost 40% of the immigrants reported a higher level of drinking than in their country of origin [14].

In the United States (US) adolescents of Chinese origin were less likely to be drinkers then Chinese adolescents in Hong Kong and American adolescents in the United States [15]. Among Mexican-heritage youth (12–17 years old) living in the US, 13% of those born in Mexico reported use of alcohol, while 19% of the US-born reported such use [16]. In two studies (one among adolescents and one among those 18 years old and over) comparing alcohol use in white and Asian subgroups, the prevalence of increased drinking behaviour ranked highest for whites, followed by Japanese or Filipinos, Koreans, Chinese and Vietnamese. Other studies show somewhat different ranks for people of Asian background [17]. Numerous studies have been carried out on alcohol use and misuse according to race/ethnicity in the US, but the country of origin is seldom included since this requires very large or country-specific samples [18]. Finally, in Australia, Vietnamese were found to consume alcohol at a lower rate than that of Australians in general [19].

Thus, alcohol use among immigrants to Western countries tended to be less prevalent among non-Western immigrants, but more prevalent and more frequent among those residing in the host country for a longer period, i.e., among second-generation immigrants (as compared to first-generation). In addition those immigrants who spoke the language of the receiving country drank more often [11,15,20-22].

Drinking is strongly influenced by other drinkers in a person’s personal network. It has been argued that society at large, or at least large segments of society, behave collectively regarding drinking [23]. A higher level of contact with one’s own dry culture will thus predict a lower level of drinking, while a higher level of contact with the host’s ‘wet’ culture will predict a higher level of drinking. Immigrants from dry alcohol cultures may influence alcohol use in the whole population in a ‘wetter’ host country, however, not only by being a low-consumption group themselves. In general, the interaction between the host society and immigrant populations has an impact on the attitudes, values and behaviours of both collectives [24]. In Norway and the Netherlands it was found that the higher the percentage of Muslims in a school, the lower the frequency of drinking both among immigrant youth and native youth from the host country [7,12]. A higher level of contact with a ‘drier’ drinking culture may thus reduce drinking also in the host population.

The pattern of drinking in Norway has been characterised by relatively infrequent consumption, but with a high level of consumption and drinking into intoxication, especially on festive occasions [25]. Alcohol sales increased by 40% from 1995 to 2009, but sales per capita in Norway were still the lowest of the Nordic countries. In 2010 sales per capita in Norway were almost half of sales in Germany, Spain and France. In a population survey from 2008 (15 years and above), 86% of women and 92% of men reported drinking during the last 12 months, while 16% of women and 27% of men reported drinking several times a week. Drinking alcohol every day with meals or in other contexts was thus not common; most alcohol was consumed on weekends [26]. There has been a reduction in drinking among young people in Norway in recent years, but still consumption and drinking into intoxication are widespread. Among 15-16-year-olds in Norway 63% of the boys and 70% of the girls reported in the 2007 ESPAD study that they had used alcohol last year, both figures at the lower end of European measurements. Boys usually drank more on each occasion than girls. Rated by the proportion of drinking into intoxication, however, young Norwegians were found in the mid-range of the European countries [27]. Non-Western immigrants to Norway have thus encountered a much ‘wetter’ drinking culture than in their country of origin, and social events on weekends included drinking from a young age.

The aim of the Norwegian government’s alcohol policies has for many years been to reduce both individual and societal costs. The measures introduced have included imposing a monopoly for the import and sale of wine and stronger alcohol, limiting access to beer sold in shops (restricted hours of sale), imposing high taxes on all sales and disseminating information on the consequences of alcohol use. Such a system has not been given priority in the EU or in single member countries (with the exception of Finland before entering the EU and Sweden) [4]. Norwegian policies have moved in a liberal direction in recent years, however, for example by opening up for personal import of wine and stronger beverages [28].

Immigration to Norway started in the latter part of the 1960s. In 1970 1.5% of the population was registered as immigrants (born outside Norway of non-Norwegian parents or born in Norway with two non-Norwegian parents). Only 0.1% came from non-Western countries at that time (Asia, Africa, South or Central America, Turkey). In 2001, at the time of the study referred to here, comparable figures for immigrants were 6.6% in total and 3.4% non-Western at the national level and 19% in total and 13% non-Western in Oslo [29]. The third generation of immigrants from those countries who came in the late 60s to early 70s had started to enter primary school in 2001. By 2011, the proportion of non-Western immigrants had increased even further [30].

The availability of three health surveys in 2000–2002 covering adolescents, adults, and adults in the largest immigrant groups in Oslo, Norway, enabled us to study alcohol drinking among ethnic Norwegians and three of the five largest non-Western immigrant groups with Islam as the predominant religion in their home country: Iranians, Pakistanis and Turks. The first aim of this study was to describe frequency of drinking in two generations of immigrants in Oslo, comparing the result to drinking among ethnic Norwegians. The second aim was to study how frequency of drinking among adult immigrants was associated with social interaction with their own countrymen and ethnic Norwegians, acculturation factors, age, gender, socioeconomic factors and the Muslim religion.

## Methods

### Sample surveys

This analysis was based on three sample surveys included in the Oslo Health Study (HUBRO), which was conducted in joint collaboration with the Oslo City Council, the University of Oslo and the Norwegian Institute of Public Health. The study protocol was approved by the Norwegian Data Inspectorate and the Regional Committee for Medical Research Ethics.

HUBRO was carried out in the city of Oslo from May 2000 to September 2001 [31]. An invitation for participation in the health survey was sent to birth cohorts of 1924, 1925, 1940–41, 1955, 1960 and 1970 who had resided in Oslo on 31 December 1999. The postal invitation included a standardised main questionnaire and an invitation to attend a clinical examination with a second questionnaire, which included acculturation-related questions. In all 18,770 individuals participated in the adult part of HUBRO, 46% of those originally invited. More details on the sampling procedure can be found elsewhere [31]. In the analysis conducted here only persons born in 1940 to 1970 (30-60-year-olds in 2000) and persons born in Norway, the Islamic Republic of Iran (response rate 48%), Pakistan (response rate 40%) and Turkey (response rate 41%) were included. This selection regarding age was imposed to restrict the sample to the parent generation of the 15-16-year-olds (see next paragraph) and other adults who socialised with them. The selection of the three countries made it possible to study drinking among people of the Muslim faith. Missing values for frequency of alcohol drinking were 1.1% for the total sample, and higher for the three immigrant groups: 6.3% for Iranians, 29.9% for Pakistanis and 1.6% for Turks.

In addition, all 15-16-year-olds on class lists in Oslo in the period of January to April in both 2000 and 2001, as well as those who entered the school later, were invited to participate in a school-based survey. A total of 8435 students were registered in or entered schools, including those in special schools for the disabled. Thirty-one were unable to respond to the questionnaire due to impairment and 88 had moved or quit school before the survey was carried out. Thus 8316 students were eligible, among whom 7343 responded to at least one question (response rate 88%). This survey consisted of a questionnaire only. Response rates for Iranians were 82%, for Pakistanis 83% and for Turks 79%. More details on the sampling procedure can be found elsewhere [32]. Missing values for frequency of alcohol drinking were 1.2% for youth with two ethnic Norwegian parents, 1.6% for youth with one ethnic Norwegian parent, 4.1% for Iranians, 2.4% for Pakistanis and 3.7% for Turks.

A third HUBRO study, the Oslo Immigrant Health Study, was conducted among the five largest immigrant groups in Oslo in 2002 [33]. All individuals born in Sri Lanka, Turkey, the Islamic Republic of Iran and Vietnam between 1942 and 1982 were invited to participate, except for birth cohorts who had been previously invited to the HUBRO study mentioned above. A 30% random sample of Pakistanis, the largest immigrant group, was also invited. A postal invitation, a physical examination and a second questionnaire were administered in the same way as for HUBRO. In the age group 30–60 years selected for analysis in this study 7890 persons were eligible for participation (response rate 40%). The response rates for three countries selected for this analysis were: Turkey 33%, Iran 39% and Pakistan 32%. More details on the sampling procedure can be found elsewhere [33]. Missing values for frequency of alcohol drinking were 2.7% for Iranians, 11.9% for Pakistanis and 6.2% for Turks.

Item missing was at 2.2% or lower for gender and country of birth/ethnicity in the three surveys.

Three datasets were established for this study, including ethnic Norwegians and/or Iranians, Turks and Pakistanis who had reported frequency of alcohol drinking:

1. The 15-16-year-olds in 2000 in HUBRO: 5381 youth with ethnic Norwegian background, 93 Iranians, 559 Pakistanis and 105 Turks. This sample was called the Oslo youth population sample.

2. The 30-60-year-olds in 2000/2001 in a merged dataset of HUBRO and the Oslo Immigrant Health Study: 12,259 with ethnic Norwegian background and 604 born in Iran, 607 born in Pakistan, and 436 born in Turkey. Age and gender weights were used to estimate the population distribution. This dataset was called the Oslo adult population sample, constituting the parent generation of the 15-16-year-olds plus those adults who socialised with them.

3. A selection from the Oslo adult population sample called the adult immigrant sample: immigrants who had attended the physical examination and delivered the second questionnaire. This sample included 389 born in Iran, 323 in Pakistan, and 270 in Turkey who also reported acculturation variables and other relevant variables. Item non-response for acculturation variables etc. was between 1.2 and 12.9%.

### Survey variables

Alcohol use among adults varies by gender, age, ethnic or religious groups and socioeconomic status [4,34]. Analyses of differences in drinking patterns between groups of immigrants and the host population should include such variables, partly due to their independent interest and partly due to possible confounding.

### How often have you consumed alcohol in the course of the past year?

The responses were divided into four categories: weekly/monthly/less than monthly/never drank alcohol.

### Socio-demographic, socio-economic and religious variables

Included gender and age, workforce participation (no, part-time, full-time), education (number of years of education), and being a Muslim.

### Country of birth/ethnic background

The place of birth was recorded as it appeared in the Norwegian Population Register. The parents’ place of birth for the 15-16-year-olds was reported by the youth in the questionnaire. The categories by country background were based on the mother’s country of birth, while the father could be from any country other than Norway. The parents of all the Iranians were from Iran, while among Pakistanis 98% were mono-ethnic, as were 97% of the Turks.

### Aspects of acculturation and interaction among adults

Various modes of the use of language and of socialisation/affiliation are frequently used to establish measures of competence and identification regarding both one’s own and the host culture [35-38]. In the HUBRO survey for adults the following seven variables were available:

How often have you in the course of the past year:

· read a newspaper in your own language?

· read a newspaper in Norwegian?

· had a visit from a Norwegian?

· received help/support from a Norwegian?

· taken part in meetings arranged by your own countrymen?

In your opinion, how good is your knowledge of Norwegian? The first five measurements had four response categories: daily/weekly/less often/never, while the sixth had five response categories: very good/good/average/rather poor/poor.

The variable ‘Number of years since moving to Norway’ was also included in the analysis as an exogenous variable to measure the length of the acculturation process.

### Immigrant background information

Immigrants have tended to settle in Norway's capital, Oslo, where they represented almost one-fifth of the population in 2001 [29]. In the HUBRO youth survey 66% of the Pakistani youth were second generation (i.e. born in Norway by two foreign-born parents); among Turkish youth, 42%; and among Iranian youth, only 3%. Immigrants from non-industrialised countries tended to concentrate in some city areas, but no specific enclaves had been established. Table 1 shows immigrant population characteristics related to aspects of integration and participation in Norwegian society [39]. All three countries studied had low levels of recorded alcohol consumption during the period from 1970 to 2000, the period of immigration to Norway. Average recorded adult (15+ years) per capita consumption of pure alcohol 1970–2000 was 0.13 litres for Iran, 0.02 for Pakistan, and 1.19 for Turkey. The comparable figure for Norway was 5.17 litres. Unrecorded consumption is not included and thus all figures may be too low [40].

**Table 1:** Immigrant group and population characteristics in Norway

**Population characteristics**	**Pakistan**	**Turkey**	**The Islamic Republic of Iran**	**Population 2005**
Status first generation	Migrant workers	Migrant workers	Refugees	-
Main settlement periods	1970 and onwards	1970 and onwards	1986 -1990, 1997-2003	-
Marriages with non-immigrant population in 2^nd^ generation Males/females	2.3%/	4.2%/	16.6%/	-
	1.3%	3.1%	19.7%	
Number of children, 1^st^ generation	3.6	2.6	2.0	1.8
Norwegian citizenship 2005	77%	75%	66%	95.4%
Voting participation 2005	54%	43%	51%	77%
Education participation, 2^nd^ generation 16–18 years. Males/females	88%/90%	82%/79%	89%/95%	90%/92%
Work force participation. Males/females	60%/28%	59%/37%	54%/45%	73%/66%

Source: Kristin Henriksen, Statistics Norway [38].

On the host country national level, the immigrant population studied varied with respect to the status of immigration (migrant workers vs. refugees), main settlement period (indicating differences in the length of the acculturation period), tendency to marry one’s own culture members, number of offspring, Norwegian citizenship, participation in voting, participation in upper secondary school level of education and workforce participation (see Table 1).

Even though the picture is not clear-cut, Pakistanis were farther away from the ethnic Norwegians than the Turks and the Iranians regarding the factors measured (more children, higher level of marrying fellow citizens, lower level of workforce participation by women). Such differences among immigrants are also found in the sample (see Table 2). Iranians had a shorter mean stay in Norway, a lower mean age and a higher education level than the other immigrants. Pakistanis included the highest proportion of Muslims.

**Table 2:** Sample characteristics of immigrants in Oslo adult population sample by country background

	**Pakistan**	**Turkey**	**The Islamic Republic of Iran**
Gender,% female	41	42	41
Mean age (SD)	44.2 (9.4)	42.3 (7.7)	41.9 (7.1)
Muslim faith,%	96	85	51
Mean number of years in Norway (SD)	20.1 (8.2)	17.2 (8.0)	12.0 (4.4)
Mean number of years in school (SD)	10.9 (3.6)	9.4 (4.4)	14.5 (3.9)
Workforce participation male, no/part time/full time (%)	22/6/72	32/5/63	30/8/62
Workforce participation female, no/part time/full time (%)	65/19/16	49/16/35	39/20/41

### Statistical methods

Demographic, social and economic characteristics of the three immigrant groups were reported from population statistics compiled by Statistics Norway and by the samples.

Frequency of alcohol use in the parent generation, 30–60 years of age, and in the young generation, 15–16 years of age, are shown in tables for gender and country background. Chi square tests were used for testing differences in the distribution of alcohol drinking frequency for groups.

A structural equation modelling (SEM) approach was used to model alcohol drinking frequency for adult immigrants. First a factor analysis approach was used to reduce the dimensions of the six acculturation variables and establish the measurement part of the model. The factors found were used as intermediate latent endogenous variables. Exogenous variables were gender, age, Muslim faith, years of education, participation in the workforce and length of residence in Norway. The structural model was developed in AMOS (Analysis of Moment Structures) version 17.0 run within the IBM SPSS Statistics version 19.0, using the modification indices approach [41]. Three goodness-of-fit measures are reported: a Chi^2^ statistic, CFI (Comparative Fit Measure) and RMSEA (Root Mean Square Error of Approximation). An acceptable fit can be based on a value of CFI higher than 0.90 and a good fit higher than 0.95 (possible range zero to one), while a value of RMSEA lower than 0.05 represents a good fit and lower than 0.08 an acceptable fit (zero is the lowest possible value) [42]. The acceptable model was run for the whole dataset using the full information maximum likelihood approach (FIML) option for imputation of missing values. This model was applied to each country, and finally a multi-group analysis was carried out to test for equality of parameters across countries.

In the final, most parsimonious model with best fit, associations between the exogenous variables and the outcome alcohol frequency can be both direct and indirect through one or more latent variables. The indirect effects are calculated as the product of any direct effect of an exogenous variable on the latent variables, multiplied by the direct effect of the latent variables on alcohol frequency. The total effect will be the sum of the direct and the indirect effects.

A significance level of 5% was used for all hypothesis tests. Few p-values for tests are reported in the text, but statements of differences are all based on tests using the 5% level.

## Results

### Comparisons of drinking

Frequency of drinking among adults was significantly different between ethnic Norwegians and all three immigrant groups, both for males and females. Among the immigrant groups, Iranians reported the highest frequency of drinking, Turks were at a middle level and Pakistanis reported the lowest frequency of drinking both for males and females (see Table 3).

**Table 3:** Alcohol frequency in Oslo adult population, by country of birth and gender

**Country of birth**	**Drinking weekly**	**Drinking monthly**	**Drinking seldom**	**Never drunk alcohol**	**Total**	**N**
Norway	54	30	14	2	100	12259
Males	62	26	10	1	100	5071
Females	48	33	17	2	100	6188
The Islamic Republic of Iran	18	30	38	14	100	604
Males	24	35	35	6	100	385
Females	7	21	44	28	100	219
Turkey	12	14	25	48	100	436
Males	19	22	30	29	100	261
Females	3	2	18	77	100	175
Pakistan	2	3	12	82	100	607
Males	4	5	21	70	100	335
Females	1	0	1	97	100	272

Age groups for ethnic Norwegians 30, 40, 45, 59–60, age groups for immigrants 30–60 years.

Comparing equality of alcohol frequency distributions, all significantly different with p < 0.000: Norwegian vs. Iranian vs. Turkish vs. Pakistani males: Chi^2^ = 3157 (12 df), Norwegian vs. Iranian vs. Turkish vs. Pakistani females: Chi^2^ = 4037 (12 df), Norwegian vs. Iranian males: Chi^2^ = 351 (6 df), Norwegian vs. Iranian females: Chi^2^ = 667 (6 df), Norwegian vs. Turkish males: Chi^2^ = 981 (6 df), Norwegian vs. Turkish females: Chi^2^ = 2481 (6 df), Norwegian vs. Pakistani males: Chi^2^ = 3081 (6 df), Norwegian vs. Pakistani females: Chi^2^ = 2481 (6 df), Turkish vs. Iranian males: Chi^2^ = 65.6 (6 df), Turkish vs. Iranian females: Chi^2^ = 98,1 (6 df), Pakistani vs. Iranian males: Chi^2^ = 343 (6 df), Pakistani vs. Iranian females: Chi^2^ = 259 (6 df), Pakistani vs. Turkish males: Chi^2^ = 117 (6 df), Pakistani vs. Turkish females: Chi^2^ = 48.1 (6 df).

Among young people, 15–16 years of age, the reported frequency of drinking was significantly lower among those with an immigrant background than among ethnic Norwegians, both for boys and girls (see Table 4.) Iranian and Turkish boys reported a higher frequency of drinking than Pakistani boys, while there was no significant difference between Iranian and Turkish boys. For girls we see the same result as for female adults. In addition, there was no significant difference between youth with two vs. one Norwegian parent.

**Table 4:** Alcohol frequency in the Oslo youth population, 15-16-year-olds, by country background and gender

**Country background**	**Drinking weekly**	**Drinking monthly**	**Drinking seldom**	**Never drunk alcohol**	**Total**	**N**
Two Norwegian parents	21	34	32	12	100	4588
Males	21	33	32	14	100	2241
Females	22	35	32	11	100	2340
One Norwegian parent	22	33	33	12	100	804
Males	23	35	28	14	100	409
Females	20	31	38	10	100	391
The Islamic Republic of Iran	12	19	32	37	100	93
Males	14	23	21	42	100	43
Females	10	16	42	32	100	50
Turkey	4	10	15	70	100	105
Males	7	15	20	59	100	46
Females	2	7	12	79	100	58
Pakistan	1	4	6	89	100	559
Males	2	6	10	82	100	287
Females	0	1	1	97	100	268

Comparing equality of alcohol frequency distributions, all significantly different with p < 0.000: Norwegian vs. Iranian vs. Turkish vs. Pakistani males: Chi^2^ = 646 (12 df), Norwegian vs. Iranian vs. Turkish vs. Pakistani females: Chi^2^ = 1051 (12 df), Norwegian vs. Iranian males: Chi^2^ = 26.7 (6 df), Norwegian vs. Iranian females: Chi^2^ = 28.7 (6 df), Norwegian vs. Turkish males: Chi^2^ = 71.3 (6 df), Norwegian vs. Turkish females: Chi^2^ = 238 (6 df), Norwegian vs. Pakistani males: Chi^2^ = 694 (6 df), Norwegian vs. Pakistani females: Chi^2^ = 1126 (6 df) For the following comparisons the p-value is given explicitly: Turkish vs. Iranian males: Chi^2^ = 3.08 (6 df), p = 0.80, Turkish vs. Iranian females: Chi^2^ = 24.7 (6 df), p < 0.000, Pakistani vs. Iranian males: Chi^2^ = 40.0 (6 df), p < 0.000, Pakistani vs. Iranian females: Chi^2^ = 162 (6 df), p < 0.000, Pakistani vs. Turkish males: Chi^2^ = 14.2 (6 df), p = 0.03 Pakistani vs. Turkish females: Chi^2^ = 30.8 (6 df), p < 0.000. Finally, the alcohol frequency distribution was not significantly different for those with two vs. one Norwegian born parent: for males Chi^2^ = 2.83 (6 df), p = 0.83 and for females Chi^2^ = 6.16 (6 df), p = 0.41.

Adult females in all groups reported a lower frequency of drinking than males (see Table 3). For adolescents there were no significant gender differences for ethnic Norwegians or group-wise for those with immigrant backgrounds. But seen as a whole, males with an immigrant background had a significantly different drinking frequency than females with the same background (Table 4).

### Analysis of drinking among adult immigrants

**Figure 1:**
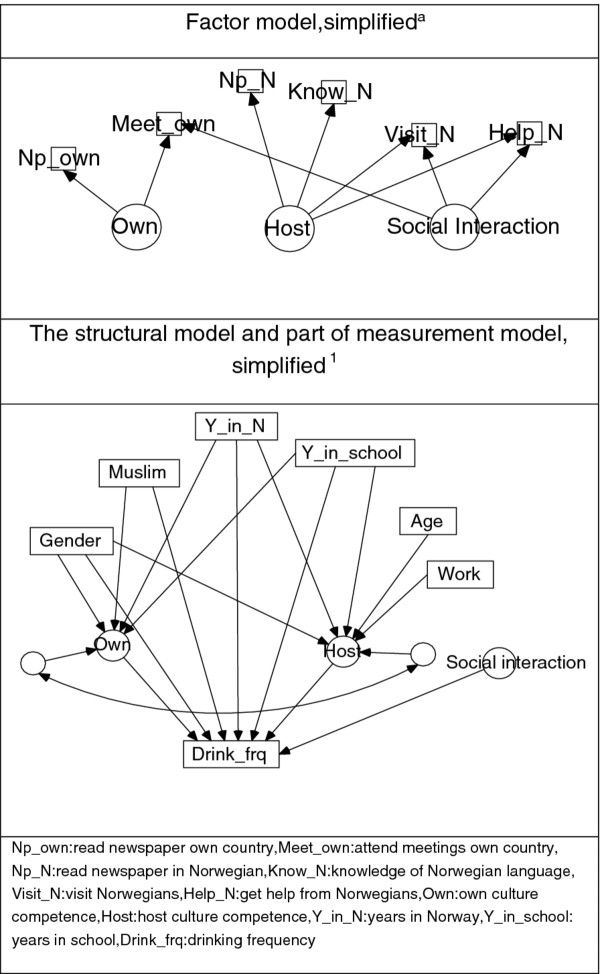
**Structural equation model for drinking frequency.** Adult immigrant sample Measurement errors, some residual terms and correlations between exogenous variables not depicted.

An exploratory factor analysis was run, which included the six variables measuring language/reading competence and social contact with own countrymen and Norwegians. It yielded three factors with eigenvalue larger than 1, explaining 75% of the variance. The variables loaded on the two first factors in a way that was expected from the literature in addition to a third factor, which was called social interaction irrespectively of own or host orientation (see also Figure 1, upper part):

1. Host culture competence (high level of Norwegian language skills/often read Norwegian newspaper/often had a Norwegian visitor/often had support from a Norwegian)

2. Own culture competence (often read newspaper in own language/often participated in meetings arranged by own countrymen)

3. Social interaction (often had a Norwegian visitor/often had support from a Norwegian/often participated in meetings arranged by own countrymen)

Rotation techniques did not improve or change the interpretation of the factors.

The structural equation model with drinking frequency as outcome (four categories) thus included six exogenous variables (gender, age, Muslim faith, years in Norway, years in school and work participation) and the three endogenous variables identified above. In the baseline model all exogenous variables could be correlated, as could the three endogenous variables. In addition all direct and indirect effects were included. Correlation between the exogenous variables in the regression on the latent variables in the structural model was highest between age and time in Norway (0.40) for the total sample. A somewhat higher value was found for this correlation for Pakistanis (0.47), while all other correlations were lower. Thus colinearity was not an issue in the regression part of the structural equation model.

Using the modification indices approach to remove all non-significant correlations and effects, an acceptable fit was obtained for the model shown in the lower section of Figure 1. Host culture competence and own culture competence mediated the association between the exogenous variables and frequency of alcohol drinking. The third factor, social interaction, did not mediate any association between the exogenous variables and alcohol frequency, but the variable’s presence was decisive for acceptability of the model. The CFI dropped to 0.75 when the social interaction factor was excluded from the model. Thus this factor had an overall association with frequency of drinking, regardless of group characteristics. Host culture competence and social interaction were positively associated with drinking frequency, while own culture competence was negatively associated with drinking frequency. Own and host culture competence were positively correlated (0.295, p < 0.05), while social interaction was not correlated with those. The regression weights, etc., are shown in the first column of Table 5.

**Table 5:** Regression weights with standard error, correlation and fit measures in adult immigrant sample, by country background

**Regressions**	**All**	**The Islamic Republic of Iran**	**Pakistan**	**Turkey**
	N = 982	N = 389	N = 323	N = 270
Alcohol frequency				
Host culture competence^1^	0.293 (0.061)*	0.175 (0.108)	0.244 (0.077)*	0.240 (0.122)*
Own culture competence^1^	−0.409 (0.103)*	−0.055 (0.066)	−0.476 (0.164)*	−0.219 (0.207)
Social interaction^1^	0.190 (0.066)*	0.176 (0.086)*	0.089 (0.101)	0.466 (0.154)*
Gender^2^	0.678 (0.068)*	0.653 (0.090)*	0.356 (0.087)*	0.913 (0.156)*
Muslim faith^2^	−0.577 (0.071)*	−0.104 (0.086)	−0.831 (0.181)*	−0.503 (0.157)*
Years in Norway^1^	−0.013 (0.004)*	0.026 (0.010)*	−0.006 (0.005)	0.003 (0.007)
Years in school^2^	0.028 (0.009)*	0.019 (0.013)	0.012 (0.015)	0.046 (0.017)*
Host culture competence				
Gender^2^	0.117 (0.046)*	−0.018 (0.057)	0.196 (0.101)	0.212 (0.089)*
Age^2^	−0.030 (0.003)*	−0.025 (0.004)*	−0.039 (0.005)*	−0.034 (0.006)*
Years in Norway^1^	0.034 (0.003)*	0.043 (0.007)*	0.054 (0.006)*	0.036 (0.006)*
Years in school^2^	0.083 (0.005)*	0.049 (0.008)*	0.098 (0.012)*	0.089 (0.011)*
Work participation^1^	0.240 (0.026)*	0.182 (0.033)*	0.230 (0.053)*	0.256 (0.049)*
Own culture competence				
Gender^1^	0.366 (0.060)*	0.282 (0.096)*	0.214 (0.101)*	0.621 (0.113)*
Muslim faith^1^	0.136 (0.068)*	−0.103 (0.093)	0.128 (0.217)	−0.043 (0.161)
Years in Norway^1^	0.011 (0.004)*	−0.001 (0.011)	0.012 (0.006)*	−0.004 (0.007)
Years in school^1^	0.015 (0.007)*	0.021 (0.012)	0.064 (0.013)*	0.046 (0.013)*
Correlation own and host culture competence^2^	0.295*	0.060	0.534*	0.516*
Model fit				
Chi-square	145*	92*	91*	89*
CFI	0.959	0.930	0.947	0.936
RMSEA	0.053	0.059	0.064	0.069

^1^ Non-variation (similarity in parameter values) between country background not rejected.

^2^ Non-variation rejected ^*^Significant at five percent level.

Being male and having attended school for many years were positively associated with drinking frequency, while being a Muslim and having lived in Norway for a long time were negatively associated with this outcome. Those having lived in Norway longest were mainly Pakistanis with low alcohol consumption. No direct association was found between age and drinking frequency, or between work participation and drinking frequency. Indirectly, however, there were associations between those variables and alcohol frequency through host culture competence. Age was negatively associated with host culture competence (−0.030 (se 0.003)), which was positively associated with alcohol frequency (0.293 (se 0.061)) and thus a total negative association was established between age and drinking frequency. Work participation was in the same way positively associated with alcohol frequency.

In addition to the direct negative association between the Muslim faith and drinking frequency, an additional negative association was mediated through a higher level of own culture competence. In addition, the positive association between the number years attending school and frequency of drinking was strengthened by the mediation through own and host culture competence. Regarding gender and years in Norway, the indirect association mediated by host and own culture competence implied a reduction of the direct effects.

When country-specific analyses were run, the CFIs were somewhat reduced, while the RMSEAs were somewhat increased for each country – indicating a poorer, but acceptable fit for each country. The same structural model was applied for the three countries, i.e. the non-significant parameters were not removed. Fewer significant results were present in the country-specific analyses, as seen by the lower number of significant coefficients in columns 2–4 in Table 5. As the fit for each country was acceptable, a multi-group analysis for country background was conducted. The measurement part of the model (weights and means) was assumed equal for each country as non-variation of this part of the model could not be rejected (Chi^2^ = 25.7 for weights with 18 degrees of freedom, p = 0.11 and Chi^2^ = 19.9 for means with 14 degrees of freedom, p = 0.13 in a nested model).

Non-variation was rejected for the rest of the model structure, however, indicating significant differences between country backgrounds (Chi^2^ = 372.2 with 26 degrees of freedom, p < 0.00). Differences in parameters between countries were identified for the association between alcohol frequency and the three variables gender (Chi^2^ = 22.2 with 2 degrees of freedom, p < 0.00), being a Muslim (Chi^2^ = 20.2 with 2 degrees of freedom, p < 0.00) and years in school (Chi^2^ = 6.7 with 2 degrees of freedom, p = 0.035). For gender the association (direct and indirect) was strongest for Turks and weakest for Pakistanis (see columns 2–4 in Table 5). Thus differences in drinking between males and females were greatest for Turks and smallest for Pakistanis when controlled for the other variables included. Being a Muslim showed the strongest positive association for Pakistanis, while no association was found for Iranians. Thus being a Muslim among Iranians did not imply differences in frequency of drinking from non-Muslims. Finally, for years in school the strongest association with alcohol drinking was found for Turks, and no association was found for the two other countries (see columns 2–4 in Table 5).

Differences in the structural model were also identified for the regression weights for the exogenous variables on the latent variables. Years in school on host culture competence (Chi^2^ = 8.7 with 2 degrees of freedom, p = 0.013) and age on host culture competence (Chi^2^ = 26.1 with 2 degrees of freedom, p < 0.00) were lowest for Iranians. Gender on own culture competence (Chi^2^ = 6.4 with 2 degrees of freedom, =0.041) was highest for Turks. In addition, the correlation between host and own culture competence was significantly different (Chi^2^ = 7.5 with 2 degrees of freedom, p = 0.023). This correlation was lowest for Iranians.

Among Iranians, direct associations with drinking frequency were found for gender and years in Norway (see Table 5). In addition, social interaction was associated with drinking frequency. An indirect association through own culture competence for gender and indirect associations through host culture competence for years in Norway and years in school, age and work participation could have been present. But own and host culture competence had no significant association with frequency of drinking for Iranians, and thus indirect associations could not be stated.

Among Pakistanis, direct associations with drinking frequency were found for gender and being a Muslim. Several indirect associations through own and host culture competence were also present, as the associations between those variables and drinking frequency were significant. There were no significant associations between social interaction and frequency of drinking (see Table 5).

Among Turks, direct associations with drinking frequency were found for gender, being a Muslim and years in school. Several indirect associations through host culture competence were present, as the associations between this variable and drinking frequency were significant. Own culture competence was not significantly associated with frequency of drinking. There was also a significant association between social interaction and frequency of drinking (see Table 5).

## Discussion

### Summary of main findings

Adults and youth with an ethnic Norwegian background reported more frequent alcohol drinking than immigrants with a background from Iran, Turkey and Pakistan. Iranians reported a higher drinking frequency than Turks and Pakistanis. For the three immigrant countries high host culture competence and social interaction were associated with a higher drinking frequency, while high own culture competence was associated with a lower drinking frequency. Muslim immigrants reported significantly lower drinking frequency than non-Muslims, and those who had lived for a longer time in Norway and were of a higher age drank less frequently. Those with a higher level of education and work participation drank more frequently.

### Multi-ethnic comparisons

Iranians had the shortest mean stay in Norway (almost all of the 15-16-year-olds were first-generation immigrants), but a higher level of drinking than Pakistanis and Turks. They were closest to the population characteristics of the Norwegian population (Table 1). Direct associations with drinking frequency were found for gender and years in Norway. In addition, social interaction was associated with drinking frequency, while own and host culture competence were not. Iran had the lowest proportion of Muslims (50%). As there was no significant difference in drinking for Muslims and others among Iranians, this did not have any impact on drinking frequency. A possible interpretation is that the Koran’s prohibition of alcohol is not obeyed to the same extent among Iranians as by immigrants from the other two countries. Iranians also had the highest average number of years in school, but this variable also lacked a significant association with drinking frequency. As the variable ‘years in Norway’ was positively correlated with drinking, Iranians may not have the same strict social norms regarding alcohol consumption as the Pakistanis and Turks, and are thus more prone to increase their drinking in a ‘wetter’ society [43]. The insignificant difference in drinking frequency between Muslim Iranians and other Iranians is an exception [7,9,10,12,44-46]. Donath et al. found that adolescents from Islamic countries in Germany had lower lifetime prevalence of drinking than German and Western European adolescents, except for students from Iran [11].

Pakistanis had the longest mean stay in Norway, and the lowest level of drinking. The second generation in Norway often married a cousin or other close family member from Pakistan. Thus the family-oriented Pakistani culture and drinking pattern has continued in Norway. Direct associations with drinking frequency were found for gender and being a Muslim. Several indirect associations through own and host culture competence were also present, and thus such culture competences played a larger role among Pakistanis than among Iranians. The general latent variable ‘social interaction’ did not play a role in relation to drinking, however. The high percentage of persons of Muslim faith (96%) and the low level of education may have strengthened the Muslim imperative to abstain from alcohol.

Turks were a group in the middle position for almost all measurements: population characteristics (Table 1), sample characteristics (Table 2) and drinking frequency (Tables 3 and 4). The recorded level of alcohol consumption in Turkey was higher than in Pakistan and this may have played a role. Direct associations with drinking frequency were found for gender, being a Muslim and years in school. Several indirect associations between exogenous variables and drinking through host culture competence were present, albeit not through own culture competence. For Turks, taking part in the host culture was thus more important for drinking than taking part in their own culture. The small number of cases in which Turks married non-immigrants may have kept the Turkish cultural expressions at a high level, as it did among Pakistanis, but the impact this had on drinking was lower. Also, a significant positive association between social interaction and frequency of drinking was present. The fact that the proportion of Muslims was high (85%) played a role.

### Acculturation and socio-demographic factors

Young immigrants of the first and second generations from Iran, Pakistan, and Turkey drank less than ethnic Norwegians, as did adults from the same countries. This is in line with earlier studies among Turks, Moroccans, Antilleans, Surinamese, Asian Muslims, Hindus, Sikhs, Vietnamese in Europe [5-12], those of Chinese origin or Mexican heritage, Japanese, Filipinos, Koreans and Vietnamese in the US [15-17], and Vietnamese in Australia [19]. Although prevalence of alcohol use and frequency of drinking have been generally lower among non-Western immigrants to Western countries, alcohol-related problems may be higher than or equal to such problems in the host population [9,47,48].

Pakistani and Turkish youth adhered to the low drinking frequency of the first generation of adults. In an earlier study in Oslo, young immigrants from Morocco, Pakistan, Turkey and Vietnam reported higher frequency of use in the second generation than in the first, by country and gender, although this was not significant [12]. In the Netherlands, Turkish and Moroccan immigrants reported less alcohol use than the Dutch population in both the first and the second generations, and their alcohol consumption did not converge towards the higher rates in the host population [5,6]. There may be an increase in drinking over time in the host country, but it will take more than two generations before differences disappear between ethnic Norwegians and groups with most non-Western immigrant backgrounds, especially when a high percentage of them are Muslims.

Lower consumption of alcohol among females than males is a worldwide finding. The exception for Norwegian adolescents seen here has been recognised in other studies [27]. The positive association between frequency of drinking and both years of education and work participation (indirectly by host culture competence) is also in line with earlier studies [4,34].

Time in the host country has been used as an indicator of integration or assimilation for immigrants. In this study drinking level was negatively associated with both mean stay in Norway and age. This reflects the fact that those with the longest mean stay were older, low-consuming Pakistanis and Turks. Country-wise analyses showed a positive association between drinking and mean stay only among Iranians. Thus the use of length of stay as an indicator of immigrant behaviour becoming closer to the host behaviour was group-dependent and not dominant.

### Social interaction

The latent variable called ‘host culture competence’ loading on the four variables 1) Norwegian language skills, 2) reading of Norwegian newspapers, 3) had Norwegian visitors and 4) had support from Norwegians, also had a social interaction component: knowledge of the host language was necessary for reading and for socialising with the host community. The variable ‘own culture competence’ loaded on two variables: 1) reading newspaper in own language and 2) participating in meetings arranged by own countrymen. Thus this variable also included a social component. The variables were positively correlated, indicating that the same person tended to score high (or low) on both variables. Thus host and own culture competence could not be measured as opposite values along one dimension only. The direction of the association with drinking was as predicted for these factors: high host culture competence was associated with a higher frequency of drinking, while high own culture competence was associated with a lower frequency. The third latent variable, social interaction, loaded on the social interaction variables mentioned above (see also Figure 1). The variable did not mediate any association from the exogenous variables on drinking and was not correlated to the two other latent variables, but was necessary for acceptance of the model. The association with drinking was positive for all immigrants, as well as country-wise for Iran and Turkey. This means that social interaction is a common quality, which in the Norwegian setting was linked to a higher level of drinking even when host and own culture competence, which imbedded social aspects, was included in the model. This is in line with Skog’s theory based on social interaction, that people will behave collectively with regard to drinking [23,49].

### Consequences of low level of drinking among immigrants

The existence of large immigrant groups of Muslim low-level alcohol consumers in Western cities contributes to a lower level of consumption at the population level and thus to lower levels of alcohol-related harm and disease in the population. First, the contribution is due to a low level of alcohol use among immigrants themselves, which applies to both the first and the second generation. The distance in drinking level to ethnic Norwegians is so long that it may take several generations to reach the host level for two of the three country backgrounds studied here. Second, a low level of drinking among immigrants who interact with ethnic Norwegians may influence the hosts to reduce their level of drinking. Dominguez and Maya-Jariego point out that interaction between members of both immigrant and host groups has an impact on the attitudes, values and behaviours of both collectives [24]. Earlier studies among young people showed that a high percentage of Muslim immigrants were associated with reduced drinking also among young people from the host background [7,12]. A similar study has not been conducted among adults. Based on the lasting low level of drinking among non-Western immigrants, the associations between drinking and the three factors own and host culture competence and social interaction found here, and also theoretical effects of social interaction on drinking, we can expect to observe reduced drinking also among ethnic Norwegians in the future.

### Weaknesses

Self-reported measures of alcohol use have demonstrated reasonable levels of reliability and validity, but many factors may influence such reports [50]. One such factor is the social desirability of drinking, which presumably is lower for persons with a ‘dry’ immigrant background compared to persons with a Norwegian background. On the other hand, in most surveys of alcohol consumption reporting of 40% - 60% can be assumed, but we do not know whether this varies by ethnicity [51]. Thus the distance from the ‘true’ difference in drinking vs. the measured one is difficult to ascertain.

Response rates for the youth part of the HUBRO were satisfactory (almost 90%), while they were much lower for the adult cohorts (46%) and the immigrant survey (40%). These were response rates based on the whole population of Iranians and Turks, not response rates for a drawn sample from those populations. In the immigrant survey, a 30% sample was first drawn of Pakistanis. Self-selection may be very important, however. Self-selection according to socio-demographic variables was shown to have little impact on several prevalence estimates in the adult survey and social inequality in health by different socio-demographic variables seemed unbiased [52]. A similar investigation of self-selection has not been conducted for the immigrant study, however. Since the setting was the same, it is plausible that the situation could be similar. Analyses that particularly addressed immigrant groups supported this [53].

Cross-sectional studies have limitations and shortcomings in the study of longitudinal social processes. The result of the acculturation process on drinking was measured at the same time as the factors assumed to influence the drinking. This is not a major problem if socialisation patterns are stable, but it is relevant to assume that such patterns develop during the acculturation process. It is a huge challenge to establish prospective studies in immigrant samples, however. It is also seldom that a cross-sectional sample as large as the one used here can be established among immigrants. Even in this study, however, some country-specific results were in line with results for the total sample, but not significantly so. This may partly be a result of less power due to a lower number of observations for each country’s background.

The three surveys employed here were conducted in 2000–2002, after 30 years of steady immigration from non-Western countries. During the 10–12 years since then, there has been a further increase in immigration from non-Western countries, and the country background has been extended to Somalia, Iraq and others. Thus the research questions addressed here have not become less topical.

## Conclusions

The existence of large immigrant groups with a low frequency of alcohol drinking in Oslo contributed to a lower level of consumption at the population level. Social integration and own and host competence were associated with drinking, as were being a Muslim, years in school and gender. Results were specific to the particular country backgrounds, however. Based on the lasting low level of drinking among non-Western immigrants, the established associations between own and host culture competence and social interaction, and also theoretical effects of social interaction on drinking, we can expect to observe reduced drinking also among ethnic Norwegians in the future. This may imply reduced alcohol-related harm and disease.

## Competing interest

There are no competing interests. The study was financed by the Norwegian Institute for Alcohol and Drug Research.

## Author contribution

The sole author conceived the idea, carried out the analyses and wrote this manuscript.

## Author’s information

The author has worked with the topic of substance use among immigrants for several years and has published nationally and internationally plus reviewed articles on the subject for peer-reviewed journals.

## Pre-publication history

The pre-publication history for this paper can be accessed here:

http://www.biomedcentral.com/1471-2458/12/535/prepub
